# A snapshot of a pandemic: The interplay between social isolation and COVID-19 dynamics in Brazil

**DOI:** 10.1016/j.patter.2021.100349

**Published:** 2021-09-15

**Authors:** Cláudia P. Ferreira, Diego Marcondes, Mariana P. Melo, Sérgio M. Oliva, Cláudia M. Peixoto, Pedro S. Peixoto

**Affiliations:** 1 Department of Basic and Environmental Sciences, Engineering School of Lorena, University of São Paulo, Lorena 12602-810, Brazil; 2 Institute of Biosciences, São Paulo State University (UNESP), Botucatu 18618-689, Brazil; 3 Department of Applied Mathematics, Institute of Mathematics and Statistics, University of São Paulo, São Paulo 05508-090, Brazil

**Keywords:** human mobility, mobile geolocation, spatial-temporal patterns, social isolation, COVID-19

## Abstract

In response to the coronavirus pandemic, governments implemented social distancing, attempting to block the virus spread within territories. While it is well accepted that social isolation plays a role in epidemic control, the precise connections between mobility data indicators and epidemic dynamics are still a challenge. In this work, we investigate the dependency between a social isolation index and epidemiological metrics for several Brazilian cities. Classic statistical methods are employed to support the findings. As a first, initially surprising, result, we illustrate how there seems to be no apparent functional relationship between social isolation data and later effects on disease incidence. However, further investigations identified two regimes of successful employment of social isolation: as a preventive measure or as a remedy, albeit remedy measures require greater social isolation and bring higher burden to health systems. Additionally, we exhibit cases of successful strategies involving lockdowns and an indicator-based mobility restriction plan.

## Highlights

• Relations between social isolation data and COVID-19 epidemiological metrics
• Two regimes of implementation of social distancing: as a preventive measure or remedy
• Late imposition of isolation (remedy) brings a greater burden for the population
• Successful strategies are presented: lockdown and São Paulo Plan

## The bigger picture

During the coronavirus disease 2019 (COVID-19) pandemic, governments used mobility data to assess the effectiveness of social distancing policies, but is it really possible to measure the effectiveness of epidemic control measures using mobility data? In this work, we found that the relationship between mobility data and epidemic metrics is far from being simple in heterogeneous countries such as Brazil, but there are clear relations between them if other factors are taken into account. We have found two regimes under which the outcome of epidemic control measures are related to mobility data, which depend on when social distancing policies were implemented. Early implementation of social restrictions as a preventive measure leads to lower incidence peaks with an overall smaller intensity of the restrictions, while the implementation at later stages, as a remedy for high epidemic metrics, while effective, requires a greater intensity of the restrictions and may bring a greater burden to the health system.

## Teaser

Do we have to stay home to control the pandemic? What happens if we do not? These are difficult questions that researchers are trying to better understand. Using Brazilian social isolation data, we found that preventive implementation of restrictive measures requires lower levels of isolation and leads to smaller incidence peaks. If we take our chances and wait to act, social distancing as a remedy works, but requires stricter measures and brings a greater burden on the health system.

## Introduction

The importance of human mobility during the coronavirus disease 2019 (COVID-19) outbreak has been clear since the beginning of the epidemic, in Wuhan, China, in late December 2019. Spreading through the highly connected network of tourism and business cities in the world, the disease became a pandemic on March 11, 2020. As an emerging disease, very little was known about it, and, until the development of vaccines, there was no definitive scientific-based medical treatment or pharmaceutical prevention method for it. Therefore, its control was based mainly on non-pharmaceutical methods seeking either to reduce the odds of contact with an infected individual causing an infection, such as mask use and handwashing, or to avoid the contact between an infected and susceptible individual, such as social distancing, lockdown, and detection and isolation of infected individuals. While the efficacy of mask use and handwashing, for example, may be quantified under controlled circumstances^1^, the efficacy of measures aiming to diminish the contacts between individuals through the decrease of human mobility may be hard to quantify.^2^ Besides, human mobility measures such as social isolation indexes^3^, ^4^, ^5^ are often not accurate or are hard to interpret.^6^

Nevertheless, the pandemic spread has been frequently modeled by dynamical systems (deterministic or stochastic models),^7^, ^8^, ^9^, ^10^, ^11^, ^12^ which usually have as a control parameter some measure of amplification/attenuation of the disease infection rate due to different transmission scenarios of varying human contact.^13^ Human contact is indirectly measured through indices of human density, mobility, isolation, and social distancing.^13^, ^14^, ^15^, ^16^ While in theory this is well accepted and provides insights on possible outcomes of the pandemic, it is usually very difficult to quantify the effects of non-pharmaceutical interventions on infection rates,^17^, ^18^, ^19^ so most studies use *ad hoc* tuning parameters.^8^, ^9^, ^10^, ^11^, ^12^^,^^20^, ^21^, ^22^, ^23^ Hence, it is important to properly understand the relationship between disease spread and human mobility data in order to better model an epidemic.

The first case of COVID-19 in Latin America was confirmed on February 25, a traveler returning to São Paulo city from Italy.^24^ São Paulo city also reported the first death in the country caused by the disease on March 12.^25^ After that, with the epidemic evolving in Brazil, on March 20 the Ministry of Health recognized that community transmission was occurring across the country, as a strategy to ensure a collective effort and logistic and financial support for states and their populations. This was followed by the implementation of nationwide non-pharmaceutical measures, including physical distancing, social isolation, quarantine, compulsory notification, and mask use.^13^^,^^26^ Due to a lack of national coordination, the pandemic was mostly fought by the 27 states and 5,568 municipalities who took diverse, heterogeneous, and mainly non-coordinated decisions by government officials at the federal, state, and municipal levels. This led to both success and failure stories and to distinct efficacy of non-pharmaceutical measures in slowing the rate of transmission across the country, which outlines the importance of taking into account the socioeconomic, geographic, and demographic backgrounds of a region when implementing such measures.^13^^,^^27^

**Figure 1:**
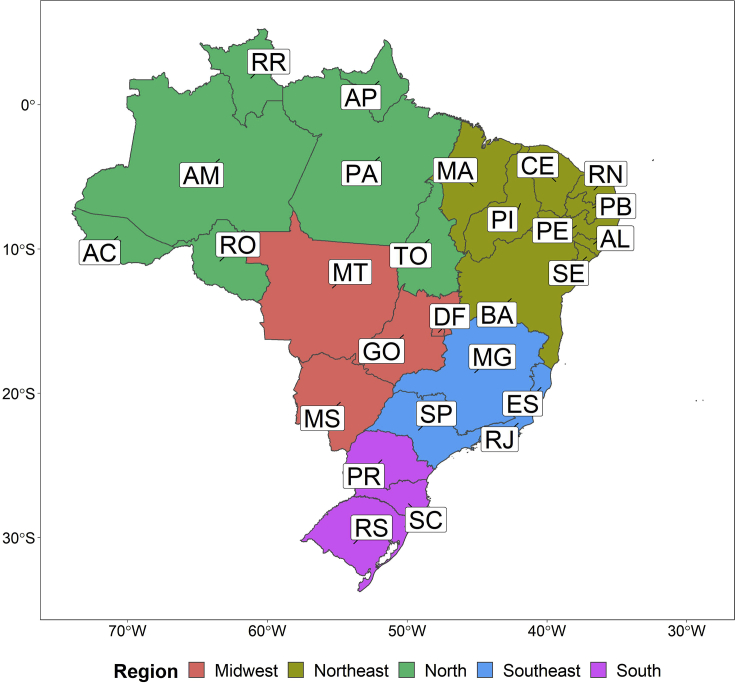
Map of Brazilian regions and states abbreviations There are 26 states in Brazil plus the DF, which holds the national capital, Brasília. They are: Acre (AC), Alagoas (AL), Amapá (AP), Amazonas (AM), Bahia (BA), Ceará (CE), Espírito Santo (ES), Goiás (GO), Maranhão (MA), Mato Grosso (MT), Mato Grosso do Sul (MS), Minas Gerais (MG), Pará (PA), Paraíba (PB), Paraná (PR), Pernambuco (PE), Piauí (PI), Rio de Janeiro (RJ), Rio Grande do Norte (RN), Rio Grande do Sul (RS), Rondônia (RO), Roraima (RR), Santa Catarina (SC), São Paulo (SP), Sergipe (SE), and Tocantins (TO).

The task of measuring the efficacy of these non-pharmaceutical interventions is especially hard in countries such as Brazil, due to its great heterogeneity.^13^^,^^28^^,^^29^ Brazil is divided into five disparate regions, shown in Figure 1. The South and Southeast regions concentrate more than half of the population, have the most developed infrastructure, are home to the wealthiest states and financial centers, offer better-paying jobs, and have better socioeconomic indexes. The Midwest region, the least populated of them, is home to the country’s capital city, Brasília, has a lower population density, contains most of the agricultural land, and has socioeconomic indexes lower than the southern regions, except for the district of the capital city, which has better indexes. The North and Northeast regions are the poorest, with lower socioeconomic indexes and underdeveloped infrastructure. Although both regions suffer from the lack of infrastructure, the situation is worse in the North, whose territory is covered by the Amazon rain forest, which makes logistics difficult in the region.^30^, ^31^, ^32^

The fast-growing literature on COVID-19 modeling, including its relations with mobility-related interventions, has tackled many issues and advanced in the understanding of the problem.^6^^,^^13^^,^^18^^,^^19^^,^^22^^,^^23^^,^^33^, ^34^, ^35^, ^36^ Regarding mobility data, mobility decrease was observed as correlated with a decrease in COVID-19 incidence in the United States.^18^^,^^33^ In Mexico, the reduction of mobility was also observed to reduce infection rates.^34^ Mobile mobility data were also used in Israel^22^ to show the effectiveness of mobility reduction in the decrease of morbidity and mortality. Similarly, mobility restrictions, observed via mobility data, had substantial effects on reducing the spread of COVID-19 in China.^23^ However, for Brazil, or other countries with similar heterogeneity, the interplay between disease transmission dynamics and control measurements that rely on mobility data is still unclear. For instance, Jorge et al.^13^ investigated the impact of governmental interventions on transmission rates for the early stages of the pandemic in Brazil (up to May 22, 2020). They use a proposed stringency index, along with mobile social distancing data, to assess the effectiveness of stringency policies enforced in Brazil, concluding that population adherence to social distancing is important for the effectiveness of the intervention. In turn, da Silva et al.^37^ used meteorological (temperature, humidity, and rainfall) and mobility data^4^ (considering a delay of 5 days) as potential predictors of daily number of COVID-19 cases (until November 6, 2020). The results are shown to strongly depend on the region in which the city is located, and only weak relations between cases and mobility social distancing data are detected. Inspired by the model proposed in Arenas et al.,^14^ Costa et al.^15^ explored different mitigation scenarios for municipalities in Brazil connected by data of inter-municipal recurrent mobility. They concluded that applying uniform mitigation measures throughout the country is not the optimal strategy, since the arrival of the epidemic at the municipalities occurs at different moments, and spreads with different velocities. Therefore, the existing literature on the matter of understanding the connections of mobility data (social distancing) and disease dynamics for heterogeneous countries such as Brazil is still very scarce and inconclusive. Hence, this work aims to unveil the complex connections between human mobility data and COVID-19 spread reduction in Brazil, taking into account many aspects of the disease dynamics, as well as the respective locations characteristics and their population, such as urban hierarchy, demography, and socioeconomic profile.

Our assessment focuses on mobility-based social isolation data, with wide coverage in Brazil,^5^ and a dataset containing daily cases of severe acute respiratory illness, considering cities in Brazil in a period ranging from March 15 to October 30, 2020. We carefully dissect this dataset, coupling its quantitative analysis with qualitative information about measures enforced to slow the disease spread and characteristics of the population and locations around the country. As a result, besides providing its own insightful knowledge of the behavior of the pandemic in different conditions, our results allow models to be more precisely adjusted to take into account local characteristics and provide more reliable epidemic scenarios. With these analyses, we hope to give a snapshot of how the pandemic evolved in Brazil and to study the interplay between social isolation and COVID-19 spread in the country.

The work is both quantitative and qualitative, and, by doing it, we were able to get some interesting insights into the dynamics of the epidemic. First, we confront the social isolation index with later daily incidence of the disease in a set of 14 large Brazilian cities, where we could assess that there is no clear relationship between the factors for all locations investigated. However, we were able to see interesting relationships when we coupled quantitative and qualitative data about the cities, taking into account both the geography and socioeconomic aspects. Then, we expanded this study to a set of 32 cities (Table 1), including capital cities and large cities with high number of cases, and identified two regimes: one in which social isolation was employed as preventive control of the disease spread, aiming to avoid an increase in incidence, which we define as a prevention regime, and another that aims to decrease an already high incidence, which we define as a remedy regime.

**Table 1:** Median of social isolation index, length of the stages (${\text{Δ}}_{i}$ with i=1,2,3), and length of the upward phase for each city

City	Median social isolation index			Length stage *i*			Length upward phase
	Stage 1	Stage 2	Stage 3	${\text{Δ}}_{1}$	${\text{Δ}}_{2}$	${\text{Δ}}_{3}$	
Aracaju	74.29	69.83	53.54	53	18	24	95
Belém	65.16	72.43	74.19	25	6	8	39
Belo Horizonte	73.39	53.45	39.33	45	35	31	111
Boa Vista	59.14	52.87	53.02	51	13	18	82
Campinas	81.63	62.20	47.93	56	26	32	114
Campo Grande	46.92	29.83	31.74	65	41	35	141
Caxias do Sul	52.47	27.07	27.14	59	31	39	129
Cuiabá	54.99	36.68	35.87	59	18	20	97
Curitiba	71.34	47.22	43.44	45	35	34	114
Duque de Caxias	60.23	56.77	58.07	23	11	18	52
Florianópolis	76.80	48.76	47.52	67	32	33	132
Fortaleza	87.73	77.72	73.76	28	11	13	52
Goiânia	54.10	42.13	38.52	88	26	46	160
Guarulhos	79.02	60.87	47.19	40	26	47	113
João Pessoa	84.72	71.22	75.23	38	14	20	72
Manaus	59.84	69.73	71.79	19	7	10	36
Mossoró	66.81	49.42	56.32	31	19	19	69
Natal	71.69	61.02	58.80	49	16	19	84
Niterói	98.67	88.17	81.82	28	14	24	66
Palmas	29.82	10.23	9.51	80	48	65	193
Porto Alegre	89.20	57.43	55.74	45	41	36	122
Porto Velho	57.38	49.35	51.82	50	17	22	89
Recife	96	89.12	92.44	24	9	14	47
Rio Branco	56.40	49.22	48.79	28	13	21	62
Rio de Janeiro	82.12	80.01	75.04	20	9	13	42
Salvador	87.71	74.16	75.11	44	16	24	84
São Luís	69.44	70.89	70.49	25	9	14	48
São Paulo	85.82	78.17	68.64	19	17	26	62
Sobral	70.06	63.69	82.71	45	10	12	67
Teresina	85.14	73.93	64.89	47	19	27	93
Vitória	87.58	73.41	62.01	30	18	23	71
Votuporanga	42.08	29.04	28.36	77	27	34	138
Quartile 1	59	49	46	28	12	18	62
Median	72	61	56	45	18	24	84
Quartile 3	85	73	72	54	26	33	114

We then turned our attention to some special cases of interest, including cities where a lockdown was effectively implemented and also taking a deeper look into the state of São Paulo, the epicenter^38^ of the epidemic and the state with better information and resources. We saw that both lockdowns and a governmental index-based plan of mobility restrictions (São Paulo Plan), which increased or decreased containment measures as the situation regarding transmission and hospital capabilities varied in regions all over the state, were effective in decreasing the incidence.

In the section “data” we present the data used in this work, while in section “results” we present the results, and in the section “discussion” we discuss our findings, the limitations of the work, and possible future works.

## Data

### Human mobility

The social isolation index is a relative measure of the number of people that do not leave their houses during the day. It can be calculated based on mobile users' mobility data from many private companies that work with geolocation services.^3^, ^4^, ^5^^,^^39^ The government of São Paulo State, in cooperation with the four main mobile network services in Brazil (Oi, Tim, Vivo, Claro), publicly provides an official social isolation index for more than 100 cities of the state. The mobile network companies use radio-based geolocation methods to infer the position of users, which limits its applicability, being adequate only for large cities with many stations. Google^4^ and Apple^3^ only provide mobility data for a small subset of Brazilian cities.

Due to the low coverage of Brazilian cities of Google, Apple, and network provider companies, in this study, we adopt a dataset provided by the company InLoco (recently the company changed name to Incognia).^5^ This company provides anonymous geolocation technologies for several mobile applications. The software development kit provided by InLoco uses multiple mobile sensors, including Wi-Fi, GPS, and Bluetooth, to infer the mobile location with an accuracy of meters. The company does not collect any user personal information and users need to *opt in* for geolocation services in the application. Several safety measures are used to ensure data safety, and the company complies with Brazilian law concerning data protection, which ensures ethical and legal assurances of data collection and usage.^40^

For the social isolation index, the home location of a user is estimated based on the recent location registered during night periods. Once the home location is known for each user in the database, InLoco calculates the number of users that left their homes during the day. The house location and the breach in isolation is calculated considering a hexagonal hierarchical geospatial indexing system (H3; https://h3geo.org/) with resolution level 8 (hexagons have edges with length approximately 460 m). The H3 data are further aggregated to city level. For this work, only aggregated city-level data with the social isolation index pre-computed by the company was available, therefore avoiding any anonymity issues concerning this work.

The main advantage of using the InLoco dataset is its wide coverage. In mid-2020, its database consisted of more than one-fourth of the mobiles in the country (due to a recent company shift in business area, the base suffered a reduction in size in 2021 and the company stopped providing the social isolation index). While these data can have sample biases, with respect to the nature of the mobile application use and smartphone diffusion in the country, it is, to our knowledge, the largest dataset of this kind for Brazil. Also, it has been widely used in the pandemic to monitor public efforts to reduce mobility and also in several academic studies.^13^^,^^36^^,^^41^, ^42^, ^43^, ^44^

In the analysis, for each municipality, the daily observed social isolation index was divided by the average value observed in the respective city from February 1 to February 15 (before carnival holiday and the start of the pandemic in Brazil). From this ratio we subtracted one and then multiplied by 100, so the relative isolation index represents the daily percentage change of isolation with respect to the mean in the first 2 weeks of February considered as a measure of isolation before the pandemic. It is important to take into account the isolation index before the pandemic, since several factors, such as the urban hierarchy, the landscape, population density, and population habits intrinsic to each city, may cause its isolation index to be higher or lower independently of any measure to increase social isolation. The transformation to percentage aids in the interpretation and understanding of the results.

### Reported cases

The number of reported cases of severe acute respiratory illness, over time, was collected from the national database of influenza epidemiological surveillance information system (SIVEP-Gripe).^45^ It comprises all severe hospitalized cases related to respiratory viruses, whose notification is compulsory in Brazil. It has been a good thermometer to catch the disease spatiotemporal dynamics in the country, since it has struggled with an insufficient capacity of molecular diagnosis and fast tests.^46^ The information available does not distinguish between imported and autochthonous cases. Although the first COVID-19 case occurred on February 25, the dataset used here ranges from March 15 until October 30, 2020, since before that changes were not yet implemented on SIVEP-Gripe to identify COVID-19 cases among cases of other diseases such as influenza, respiratory syncytial virus, adenovirus, and parainfluenza.^47^ This period comprises the first wave of the disease in Brazil, during which it is assumed that the transmission was dominated by a unique variant of the virus in Brazilian territory.^42^

A nowcasting procedure was performed, using the R package NobBS,^48^ to correct delay in notifications, which in Brazil can take up to 40 days. Since the nowcasted daily number of cases still presented weekly variations, it was smoothed by taking a 7-day moving average. To compare the disease incidence in each city, the daily number of cases was divided by 100,000 inhabitants.

The effective reproduction number ($R_{t}$) was calculated using the nowcasted smoothed data of incidence. For this, we considered the epidemiological model susceptible-exposed-infected-recovered (SEIR), and the approach proposed by Wallinga and Lipsitch.^49^ The parameters used to calculate $R_{t}$ are the latent period ($\eta^{-1}$) of 3.0 days, the infectious period ($\tau^{-1}$) of 6.4 days, and the life expectancy in Brazil ($\mu^{-1}$) of 75 years. The rates of leaving the exposed and infectious classes are denoted by $s_{1}=\eta +\mu$ and $s_{2}=\tau +\mu$, respectively. Therefore, the generation interval distribution g(t) is given by^50^

$$g\left(t\right)=\sum_{i=1}^{2}\frac{s_{1}s_{2}e^{s_{i}t}}{\prod_{j=1,j\neq i}^{2}\left(s_{j}-s_{i}\right)}\;\text{with}\;t\geq 0.$$

After normalizing g(t) we can evaluate $R_{t}$ as

$$R_{t}=\frac{b\left(t\right)}{\int_{0}^{\infty }b\left(t-a\right)g\left(a\right)da},$$

where b(t) accounts for the number of new cases at day *t*.

We highlight that all data used in this work are provided as supplementary material.

### Urban hierarchy, Human Development Index, and transportation infrastructure

One variable that can directly affect the spread of the epidemic over the country is the hierarchy of urban centers. In Brazil, the classification or urban centers is done by the Brazilian Institute of Geography and Statistics (IBGE),^31^^,^^51^ and is based on territory management, offering of trade and services, financial services, university education, media and communication markets, culture and sports, transportation services, agricultural activities, and international links. Among the 32 cities considered in this work, there are 14 metropolises and three cities belonging to their greater area, 14 regional capitals, and one sub-regional center, which are, respectively, the top three levels of the hierarchy consisting of five levels. It is important to take into account the hierarchy to better understand the timing of the disease arrival in each city.

Socioeconomic variables are also usually associated with disease spreading, and the Human Development Index (HDI) is known to be an important factor. The HDI combines measurements of life expectancy, education, and *per capita* income.^32^ It ranges from 0.418 to 0.862 among all cities in Brazil, while the state's averages ranges from 0.631 to 0.824. The index is generally greater for states in the center-south regions and lowest in the northern regions. Indeed, the highest average index can be found at São Paulo (SP), Santa Catarina (SC), and Federal District (DF), and the lowest at Pará (PA), Alagoas (AL), Maranhão (MA), and Piauí (PI). The HDI of the 32 cities considered ranges from 0.711 to 0.847.

The transportation infrastructure varies around the country and may also influence disease spread.^38^ Although the primary means of transportation is by road, it is highly concentrated in the center-south regions, especially in the state of São Paulo. The exception to this rule is the Amazon region, where road transportation loses its importance to waterways thanks to the dense natural river network. The lack of adequate roads in the North region and the logistical issues with connecting roads with waterway transportation are some of the bottlenecks of the region's development. From a human mobility point of view, while the majority of trips within states are by road, the main means of transportation between states are airplanes, with a network of around 100 airports around the country.^30^ International airports are located at São Paulo (SP), Rio de Janeiro (RJ), Brasília (DF), Belo Horizonte (MG), Campinas (SP), Salvador (BA), Fortaleza (CE), Recife (PE), Porto Alegre (RS), Florianópolis (SC), Manaus (AM), Belém (PA), Natal (RN), and Campo Grande (MS).

## Results

We start by analyzing the relationship between the isolation index and the incidence in the main cities in Brazil in the hope of better understanding how they may be related. Then, we expand the analysis and look for distinct possible relations between the isolation and the incidence for all the cities in the study. In the last sections, we focus on more specific cases where the isolation had a special influence on the incidence. They comprise cities that employed a lockdown and the São Paulo State plan to control the disease spread within the state.

### Social isolation index versus incidence in Brazil main cities

In this section, we study the relationship between the daily social isolation index and the daily incidence in the main cities, which are the metropolises São Paulo, Rio de Janeiro, Belo Horizonte, Curitiba, Florianópolis, Fortaleza, Goiânia, Manaus, Belém, Porto Alegre, Recife, Salvador, Vitória, and Campinas. The capital city of Brasília is not considered due to the lack of good incidence data. In this analysis, we consider the average of the relative social isolation index between 7 and 13 days ago ({i−13,…,i−7}) as the isolation measure of the day *i* (lag of 7 days between incidence and social isolation index). This is done since any effect of the social distancing on the incidence observed in a day should be due to the social distancing of at least 1 week before, as we ought to account for the incubation period or the serial interval (between 2 and 14 days).^22^^,^^33^^,^^52^, ^53^, ^54^, ^55^ The 7-day average ensures that specific day-of-the-week effects are removed from data. Additionally, in early stages of this study, different lag periods were analyzed and this chosen period (7–13 days of delay) provided adequate fittings, in agreement with other studies that also looked at relations between the daily number of COVID-19 cases with the human mobility^13^^,^^36^ or contact^16^ indexes.

**Figure 2:**
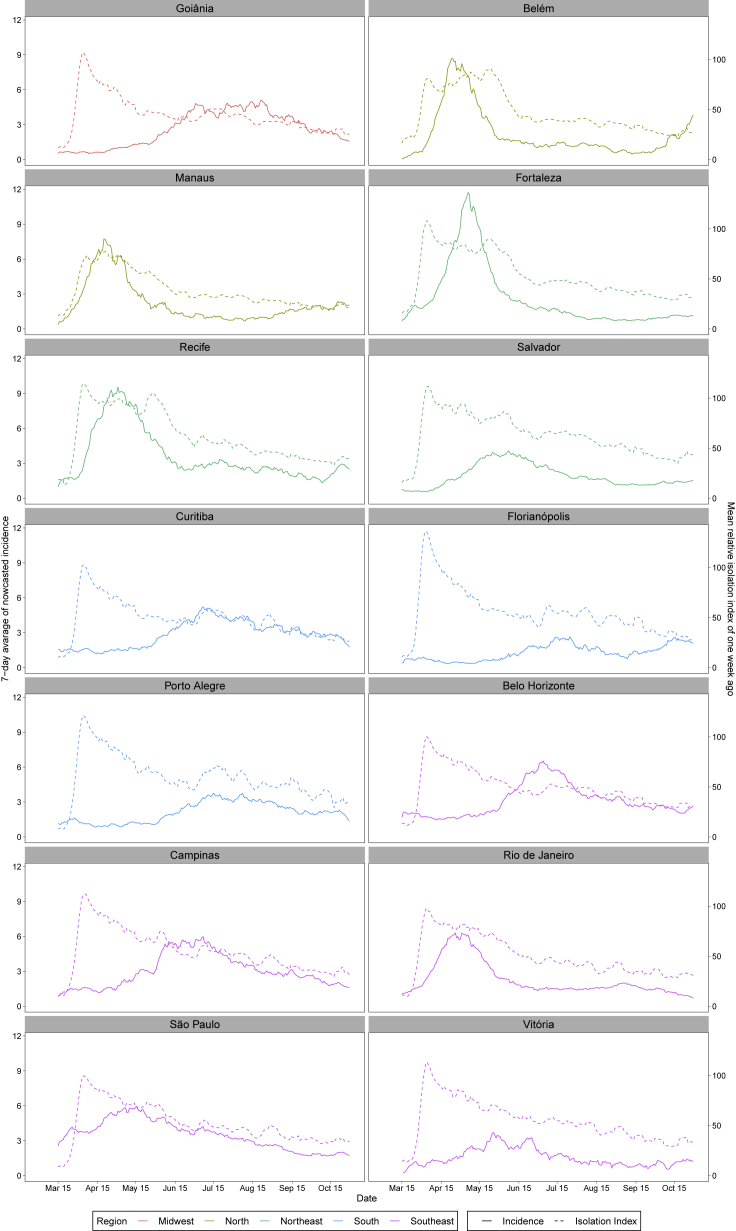
Temporal evolution of the mean relative isolation index of 1 week ago (dashed line) and the 7-day moving average of incidence (solid line) for the main cities in Brazil Colors refer to the five geographic regions that divide the country.

Figure 2 shows the daily incidence and the social isolation index for each city. Spatial-temporal variations of both measures highlight regional and temporal differences resulting from geographic, demographic, cultural, and political characteristics of each main city of Brazil, whose behavior affects other cities in their region of influence. We see that, considering all cities together, the monthly average of the social isolation index decreased with time: from April to October they were (average value and its standard deviation), respectively, 81±18;68±19;50±17;50±16;43±16;35±17; and 31±16.

**Figure 3:**
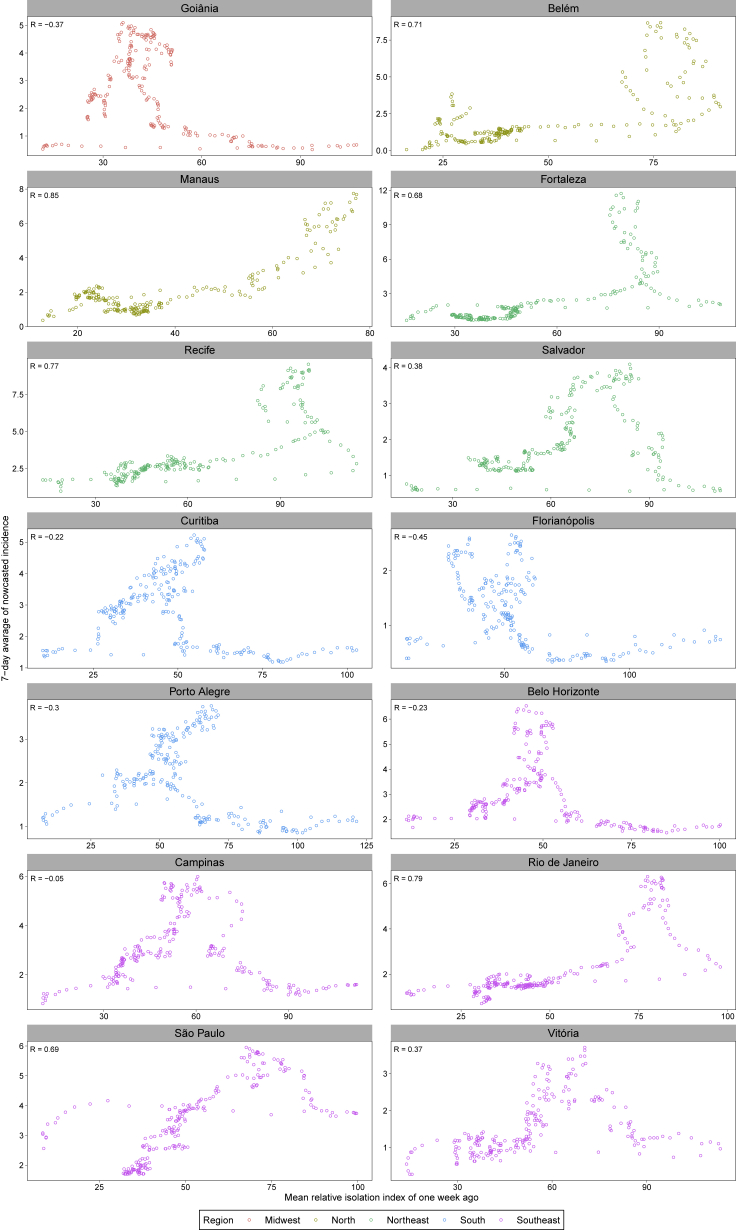
Dispersion between the mean relative isolation index of 1 week ago and the 7-day moving average of incidence for the main cities in Brazil Colors refer to the five geographic regions that divide the country. The linear correlation coefficient (R) is presented in each plot.

For some cities, we see an increase in the incidence following a decrease on the isolation (cf. Belo Horizonte, Campinas, Curitiba, Goiânia, Porto Alegre, Salvador, and Vitória), although there is no clear functional relationship between the incidence and isolation (Figure 3). Indeed, for some cases we see a positive, and in some cases a negative, linear correlation coefficient between the daily incidence and the isolation measure, highlighting the lack of a clear relationship, such as linear, satisfied in all cities. The main findings from the analysis of Figures 2 and 3 are that (1) there is no functional relationship between incidence and isolation, let alone a linear relation, and, even though this is the case, (2) there may be a relationship when we interpret the results in view of characteristics of the city and the disease spread within it, as each city has a peculiar behavior regarding the daily incidence and isolation over time. This analysis of the main cities of Brazil is the purpose of this section.

In order to mitigate the effects of the disease on the healthcare system, lockdown strategies were implemented on some cities in the North and Northeast regions, causing the sharp increase in the social isolation index measured in Belém, Fortaleza, and Recife (see Figure 2 around May 15). In all cities, the lockdown can be clearly seen in the data, especially in Recife, which is the city among the three where the strategy most increased the isolation. For these cities, the epidemic peak was from 9 to 12 cases per 100,000 inhabitants and happened prior to the lockdown.

We see in Figure 2 that, among the cities of the Northeast region, there is Salvador, which seems to have kept a more efficient control of the disease transmission, since it endured a higher isolation rate, and had a smaller peak, compared with other cities of this region. On the other hand, there is Fortaleza, which has the lowest HDI among the three Northeast cities considered and is one of the main entrance points of travelers from outside the country visiting the North and Northeast regions, factors that can explain its low performance in controlling the spread of the disease relatively to the other two cities. Likewise, in the North region, despite the fact that the social isolation index was higher at Belém compared with Manaus, both cities had a similar epidemic temporal evolution pattern. Among the cities, they are the ones with the lowest HDI and are highly connected by waterways. They belong to the Amazon region, where the spread of COVID-19 took the course of the waterways and was enhanced by the long duration of the trips in boats, where mitigation strategies, such as socially distancing and handwashing, are compromised.^56^

In the Southeast region, Belo Horizonte and Campinas had a similar temporal pattern of disease transmission. Vitória had the best outcomes on disease control transmission, probably because it is among the cities with the highest HDI and lacks an international airport. Rio de Janeiro and São Paulo are highly connected by roadways and airways and share a similar pattern of social isolation index, with São Paulo, in general, having a better outcome for controlling the disease spreading, especially at the beginning of the epidemic. Among the South cities, despite Curitiba not having an international airport, it had the worse social isolation index compared with Porto Alegre and Florianópolis, which may be behind its larger number of reported cases. It is worth mentioning that even though Florianópolis has a high HDI, the major part of its territory is on an island, which could have contributed to its good outcome in controlling the disease spreading compared with the other cities in the south of Brazil.

We see that Goiânia, the only city of the Midwest region considered, did a better job in containing the spread of the disease, compared with cities of other regions. An important factor that could have contributed to this is that the Midwest region annually records the lowest number of influenza and other respiratory virus cases inside the national territory,^57^ so Goiânia could be prone to having a lower incidence of respiratory diseases in general.

### Two regimes of disease control and the employment of social isolation as remedy or prevention

**Figure 4:**
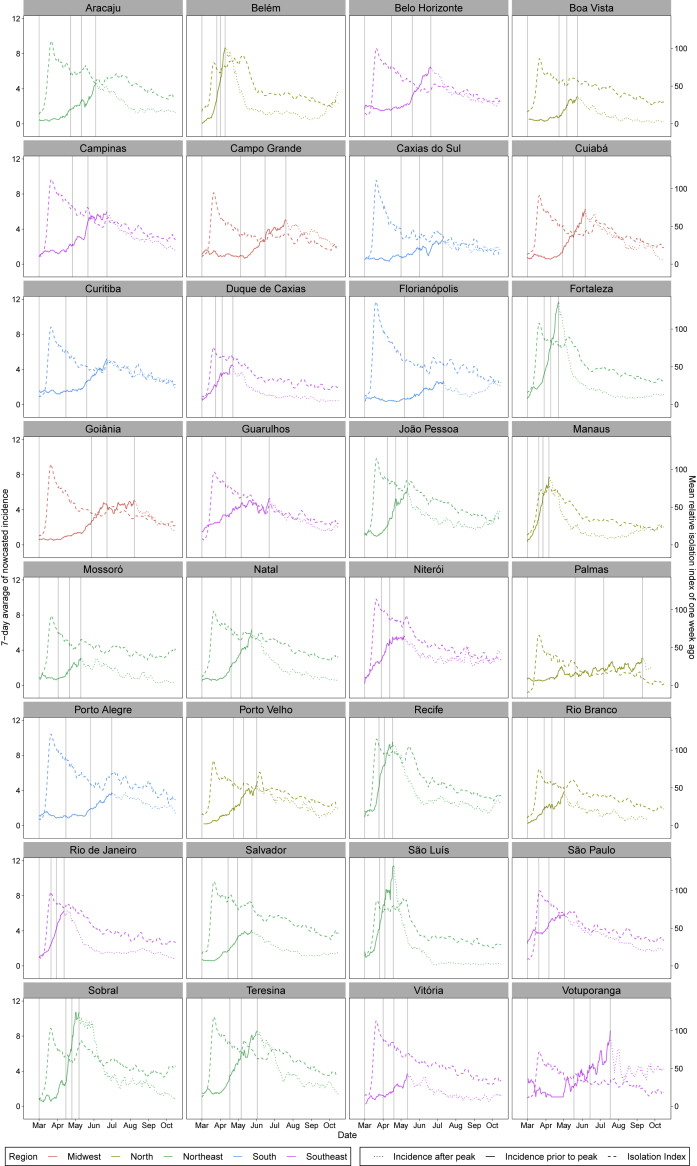
Temporal evolution of the mean relative isolation index of 1 week ago (dashed line) and the 7-day moving average of nowcasted incidence (solid line prior to the peak and dotted line after the peak) for 32 selected cities in Brazil Colors refer to the five geographic regions that divide the country. The vertical lines delimit the three stages of the upward cases, which represent 0%–25%, 25%–50%, and 50%–100% of the accumulated cases until the peak (upward phase).

In this section, we study the relationship between the social isolation index and the time to reach the peak of incidence over different geographic locations around the country. For each of the 32 cities considered, we divide the period from March 15 until the day with the maximum incidence into three stages according to the accumulated number of cases in the period. We call this the upward phase of the disease. Stage 1 goes from March 15 until 25% of the total cases of the upward phase is reached, and stage 2 goes from when 25% until 50% of the total cases of the upward phase is reached. Finally, stage 3 starts when 50% of these cases is reached and ends at peak of cases. Figure 4 presents the daily incidence, isolation index, and the limits of the three stages for each city. In Table 1 we present the length and median isolation of each stage for each city.

A small upward phase is related to a fast inversion of the incidence trending, from an increase to a decrease in the daily number of cases, while a longer phase is characterized by a longer time to reach the peak and to invert the trend of the disease. The time to invert the trend alone is not a measure of the quality of disease spread control, since the peak may be too high, so reaching it fast or taking a long time to reach it may not be relevant if sooner or later it brings the health system to a collapse. Nevertheless, in any case, a negative association between isolation and the length of these stages may be evidence that high isolation was determinant in inverting the trend of the disease (shortening the stages' lengths), independently if such a trend was at a high incidence. Indeed, we identified a pattern in the data representing two regimes of disease spread where we could see distinct impacts of the isolation index on the disease spread, which are as follows. Here, we remark that herd immunity was not reached during the period under consideration in this study in any of the cities considered, as was observed by serological inquiries done in Brazil (see, for example, Oliveira et al.,^58^ or Buss et al.^41^ for the most prominent case, of Manaus city), so that the reasons for reaching the peak in this case are mainly related to non-pharmaceutical interventions and intrinsic population contact patterns.

**Figure 5:**
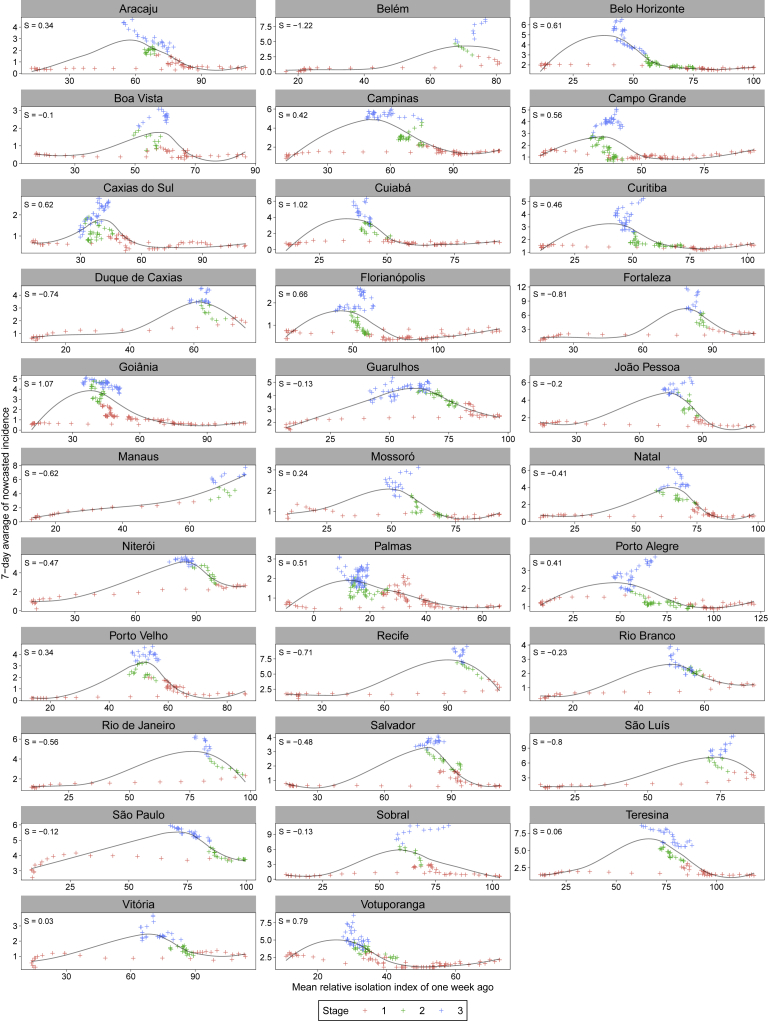
Dispersion between the mean relative isolation index of 1 week ago and the 7-day moving average of incidence for 32 cities during the upward phase. Colors refer to the stage. The line is a LOESS . The value of *S* is the skewness coefficient of the normalized LOESS curve.

In Figure 5 we present the dispersion between the daily incidence and the mean isolation index of 1 week ago for the cities considered during the upward phase. We also present the locally estimated scatterplot smoothing (LOESS) curve for each plot.^59^ We observe two distinct behaviors among the cities that are associated with the skewness of the smooth curve. There are the cities that did not have a large isolation in most of the stage 1 and had a joint increase in the incidence and the isolation in stages 2 and 3. Examples of these cities are Belém, Fortaleza, Manaus, Recife, and Rio de Janeiro. These are cities that started the employment of measures to stop the disease spread when the incidence was already high; hence, we observe a greater incidence when there was greater isolation. In these cases, the smooth curve is negatively skewed. This regime is characterized by the employment of social isolation as a *remedy* to decrease an already high incidence: isolation was increased as an attempt to stop further increase in the incidence.

The other behavior is that of cities that had a great isolation early on in stage 1, before any increase in the incidence and then, as time went by, decreased the isolation during stage 1 to a point when the incidence started to increase and stage 2 was reached. From this point, the isolation stayed stable, and the incidence increased until reaching the peak. Examples of cities with this behavior are Belo Horizonte, Florianópolis, Porto Alegre, and Votuporanga. These are cities where the measures to stop the disease started before the increase in the incidence, which could have contributed to delaying the increase that happened only when the isolation decreased. In these cases, the smooth curve is positively skewed. This regime is characterized by the employment of social isolation as a *prevention* to avoid the increase on the incidence: isolation was increased before an increase on the incidence, and avoided it while isolation remained high.

**Figure 6:**
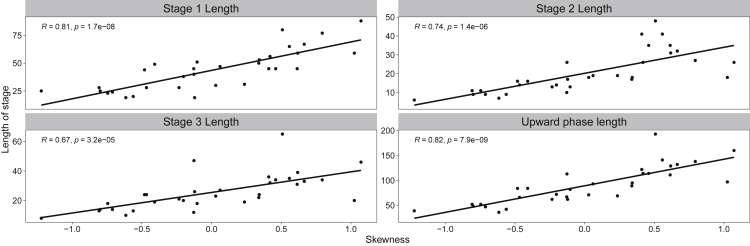
Dispersion between the length of each stage and the skewness coefficient of the smooth curve, and dispersion of the length of the upward phase and the skewness coefficient of the smooth curve. The linear correlation coefficient (R) and its p value is present on the plot.

**Figure 7:**
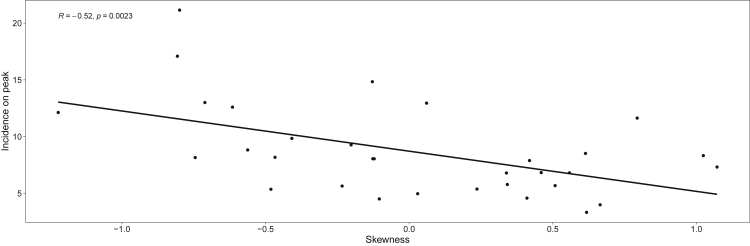
Dispersion between the incidence on the peak and the skewness coefficient of the smooth curve. The linear correlation coefficient (R) and its p value is present on the plot.

**Figure 8:**
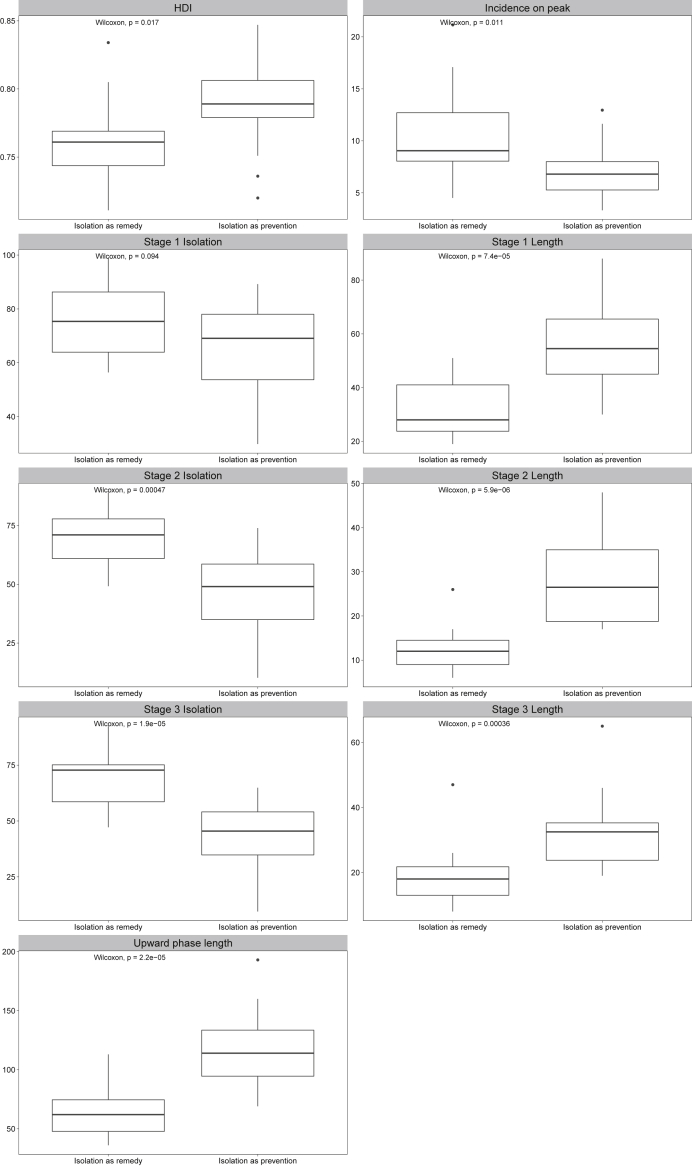
Box plots of HDI, incidence on the peak, median isolation on each stage, length of each stage, and length of the upward phase for the cities that employed isolation as a remedy (negative skewness coefficient of the smooth curve) and cities that employed it as prevention (positive skewness) p values refer to the Wilcoxon test comparing the two groups of cities.

These two regimes, characterized by positive and negative skewness of the curve in Figure 5, represent two ways that social isolation may be influencing incidence. We see in Figure 6 a positive correlation between the length of each stage and the length of the upward phase, and the skewness coefficient. Furthermore, in Figure 8 and Table 2 we see that cities with negative skewness (isolation as remedy) tended to have lower lengths of all stages compared with cities with positive skewness (isolation as prevention) but tended to have a greater isolation in all stages. This means, for instance, that cities with low (negative) skewness had faster stages, which can be due to the fact that they increased the isolation when the incidence was already high and therefore were able to rapidly change the trend of cases, but to cause such change the isolation had to be very high. In the same manner, cities with greater skewness coefficient employed the isolation early on, and kept a low incidence until a decrease in the isolation happened and was followed by an increase in the incidence; the delay in the increase of the incidence caused the stages to last longer and the isolation was not as high in all stages, decreasing from stage 1 to 3. This shows that social isolation may influence the incidence by remedying high incidence or by preventing it, although a higher isolation is needed to remedy, while lower isolation may prevent. Nevertheless, there is a negative aspect of remedying. We see in Figure 7 a negative correlation between the incidence of the peak and the skewness, and in Figure 8 and Table 2 we see that cities that remedied tended to have a greater peak.

**Table 2:** Descriptive statistics of the HDI, incidence on peak, length of each stage, length of the upward phase, and median isolation index on each stage for cities that employed isolation as a prevention and as a remedy measure

Variable	Isolation as	Size	Mean	SD	Minimum	First quartile	Median	Third quartile	Maximum
Isolation stage 1	remedy	16	75.86	13.66	56.40	63.93	75.36	86.29	98.67
	prevention	16	65.25	17.59	29.82	53.69	69.08	78.01	89.20
Isolation stage 2	remedy	16	69.75	11.69	49.22	60.98	71.05	77.84	89.12
	prevention	16	47.50	17.95	10.23	34.97	49.05	58.62	73.93
Isolation stage 3	remedy	16	69.19	12.74	47.19	58.62	72.78	75.14	92.44
	prevention	16	43.35	14.63	9.51	34.84	45.48	54.09	64.89
Length stage 1	remedy	16	31.62	11.06	19	23.75	28	41	51
	prevention	16	56.06	16.43	30	45	54.50	65.50	88
Length stage 2	remedy	16	12.56	4.84	6	9	12	14.50	26
	prevention	16	28.19	9.84	17	18.75	26.50	35	48
Length stage 3	remedy	16	18.81	9.17	8	13	18	21.75	47
	prevention	16	32.50	11.40	19	23.75	32.50	35.25	65
Length upward phase	remedy	16	63	20.53	36	47.75	62	74.50	113
	prevention	16	116.75	32.41	69	94.50	114	133.50	193
Incidence on peak	remedy	16	10.42	4.49	4.51	8.05	9.05	12.70	21.15
	prevention	16	6.92	2.58	3.33	5.28	6.80	8	12.95
HDI	isolation as remedy	16	0.76	0.03	0.71	0.74	0.76	0.77	0.83
	isolation as prevention	16	0.79	0.03	0.72	0.78	0.79	0.81	0.85

We conclude then that social isolation may be successfully employed to reduce the disease spread, either as a remedy, when incidence is high, or as a prevention, to avoid high incidence. Nevertheless, when it is employed as a remedy, the isolation should be greater and the peak of the disease tends to be higher. Hence, isolation may be effective if employed when the incidence is low and, even if a greater peak can be avoided, it must be kept high during all the epidemic, otherwise, although delayed, an increase in the incidence eventually occurs.

We observe in Figure 8 and Table 2 that the cities that employed isolation as prevention tend to have a higher HDI than cities that remedied high incidence. It is evidence that more developed cities tended to better manage the disease spread, employing isolation as a measure to avoid high incidence, being successful as long as isolation was kept high.

### Lockdown strategy

**Figure 9:**
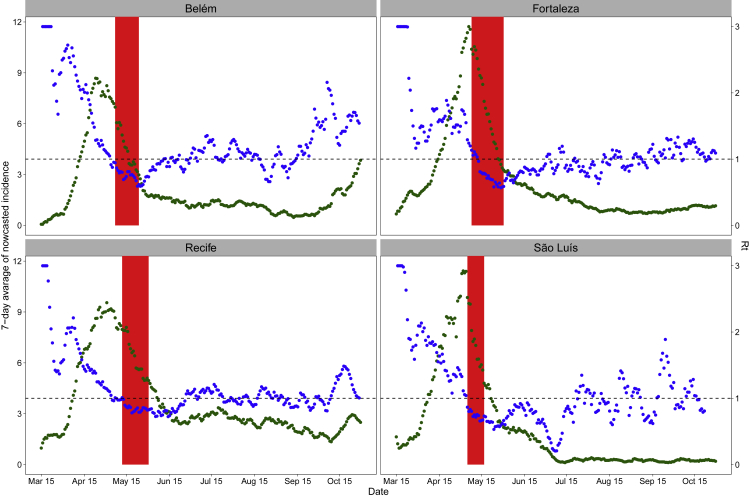
Temporal evolution of the 7-day moving average of incidence (green), and $R_{t}$ (blue) In red, we have the lockdown period. The dashed horizontal line is drawn for $R_{t}=1$. The $R_{t}$ values were truncated at three for a better visualization.

Strict lockdown strategies were not common in Brazil in 2020, but were nonetheless implemented in four capital cities of states in the northern regions. In Figure 9 we present the daily incidence and the $R_{t}$ for four cities that implemented a lockdown, namely São Luís, Belém, Fortaleza, and Recife. Among the four cities, the first confirmed case occurred in Recife on March 12, which was then followed by Fortaleza on March 15, Belém on March 18, and São Luís on March 20. The disease spread differently in all cities, which also have distinct hospital and testing capacities. The first city to declare lockdown was São Luís on May 5 when it achieved 10.75 new cases per day (average of the 7 days before lockdown). It was then followed by Belém on May 7 with 7.14 new cases per day, Fortaleza on May 8 with 11.35 new cases per day, and Recife on May 16 with 8.17 new cases per day.

In São Luís, the lockdown took 13 days, and a reduction of 34% in the number of cases per day was observed. In Belém it took 18 days with a reduction of 40% in the number of cases per day. In Fortaleza, it took 24 days, with a reduction of 39% of the cases per day. Finally, in Recife it took 16 days with a reduction of 24% on the number of cases per day. Furthermore, we see in Table 3 that, 4 weeks after the lockdown was over, the incidence decreased at least 70% compared with the week prior to the lockdown, a number that was as high as 84% in Fortaleza. The increase in the isolation during the lockdown compared with the week leading up to it varied from 4% in Belém to 20% in São Luís, although this isolation could not be maintained in the weeks after the lockdown, attaining levels lower than those observed before the lockdown in all cities.

**Table 3:** Prior: The mean relative isolation index and mean 7-day average incidence on the week leading up to the lockdown in each city

Belém			Fortaleza			Recife			São Luís		
Period	Var. Isol.	Var. Inc.	Period	Var. Isol.	Var. Inc.	Period	Var. Isol.	Var. Inc.	Period	Var. Isol.	Var. Inc.
Prior	82.05	7.62	Prior	75.32	11.17	Prior	83.54	8.38	Prior	73.43	10.86
Lock	0.04	−0.40	Lock	0.09	−0.39	Lock	0.15	−0.24	Lock	0.20	−0.34
1	−0.30	−0.73	1	−0.23	−0.73	1	−0.15	−0.48	1	−0.17	−0.61
2	−0.46	−0.77	2	−0.34	−0.78	2	−0.24	−0.61	2	−0.28	−0.75
3	−0.48	−0.79	3	−0.41	−0.80	3	−0.33	−0.67	3	−0.43	−0.81
4	−0.54	−0.80	4	−0.39	−0.84	4	−0.27	−0.71	4	−0.46	−0.82
5	−0.52	−0.83	5	−0.37	−0.84	5	−0.32	−0.67	5	−0.53	−0.84
6	−0.51	−0.85	6	−0.36	−0.85	6	−0.36	−0.67	6	−0.51	−0.88
7	−0.53	−0.84	7	−0.38	−0.86	7	−0.33	−0.64	7	−0.46	−0.93
8	−0.54	−0.82	8	−0.37	−0.87	8	−0.36	−0.62	8	−0.49	−0.98
9	−0.50	−0.83	9	−0.41	−0.90	9	−0.39	−0.67	9	−0.50	−0.98
10	−0.52	−0.84	10	−0.50	−0.92	10	−0.48	−0.69	10	−0.47	−0.97
11	−0.60	−0.82	11	−0.45	−0.92	11	−0.44	−0.70	11	−0.49	−0.97
12	−0.54	−0.84	12	−0.48	−0.92	12	−0.45	−0.72	12	−0.57	−0.97

Lockdown (lock): proportional variation in the mean isolation index and mean 7-day average incidence during the lockdown compared with the mean observed in the week leading up to the lockdown. Periods: proportional variation (Var.) in the mean isolation index (Isol.) and mean 7-day average incidence (Inc.) during the first to tenth week after lockdown compared with the mean observed in the week leading up to the lockdown.

### São Paulo mitigation strategies

The history of COVID-19 spreading in the state of São Paulo is characterized by different temporal and spatial scales. Starting from the regional centers, the disease displaced to municipalities with major connections, and later to municipalities with minor connections, and lastly to rural municipalities (spatial pattern well explained by the gravity model). Locally, the spread was by contiguity (diffusion model).^60^ As the disease spread, following the example of the metropolitan region of São Paulo, the inner state adopted strong mitigation measures, closing schools, universities, and all its trade, keeping only essential services such as pharmacies, supermarkets, and hospitals open. This delayed the arrival of the virus to the inner state by at least 1 month, letting the cities prepare their healthcare systems, which are more fragile in this region.

On May 27, São Paulo State started to move out from a restrictive quarantine with a flag system that classified, based on several indicators, the disease transmission risk, and the probability of break-down of the healthcare system. Five colors were adopted: (1) red is a contamination phase and only essential services are permitted; (2) orange is an attention phase, with the possibility of some services opening; (3) yellow is a controlled phase, with some flexibilization; (4) green is a partial opening phase, in which all services are allowed to open; and (5) blue has only restrictions over events that generate large agglomerations of people (São Paulo Plan; https://www.saopaulo.sp.gov.br/planosp/). These phases were attributed to each region of the state, defined according to a division of its healthcare system.^61^ These 17 regions, called Regional Health Departments (DRS in Portuguese), are each represented by a major centralized city.

**Figure 10:**
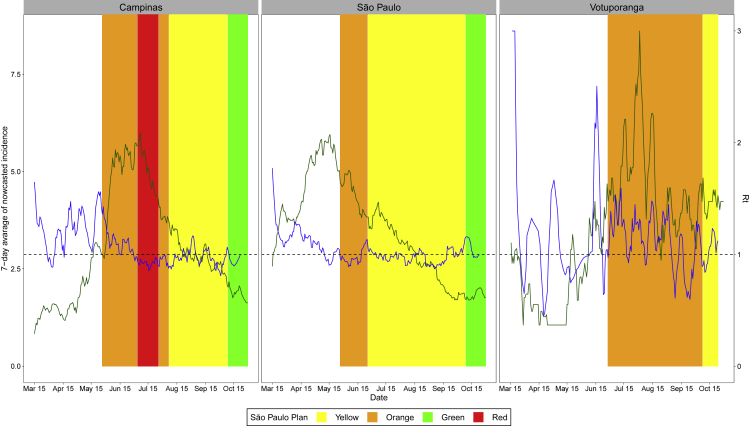
São Paulo (DRS I: São Paulo), Campinas (DRS XII: Campinas), and Votuporanga (DRS XV: São José do Rio Preto) Temporal evolution of the 7-day moving average of incidence (green) and $R_{t}$ (blue). The colored vertical bands represent the phases of São Paulo Plan. The dashed horizontal line is drawn for $R_{t}=1$. The $R_{t}$ values were truncated at three for a better visualization.

Figure 10 shows the temporal evolution of the incidence and the $R_{t}$ for three cities in São Paulo State: the capital city São Paulo; the most important city in the inner state, Campinas; and the city of Votuporanga, which is a sub-regional center under the influence of São José do Rio Preto, a regional capital. The colors (vertical bands) are associated with each moment of the São Paulo's Plan in each city. The peak of the incidence curve occurred on May 1 at São Paulo (with eight new cases), on June 10 at Campinas (with 7.8 new cases), and on August 10 at Votuporanga (with 11.6 new cases). This pattern shows the spread of the disease from the metropolis of São Paulo to inner cities in a lower degree of urban hierarchy. Besides, less complex cities (lower level of urban hierarchy compared with São Paulo) were not able to achieve a high social isolation index or keep it for a long time (see Figure 4). The variation observed in the transmission rate can be related to the population density in each city, which are respectively 7,398.3, 1,359.6, and 201.2 inhabitants/km^2^ at São Paulo, Campinas, and Votuporanga. Although the incidence was higher at Votuporanga, the average absolute number of cases was 3.5 (from 1 to 11), 36.5 (from 9 to 95), and 428.2 (from 161 to 986) in Votuporanga, Campinas, and São Paulo, respectively.

In Table 4 we present the median $R_{t}$, nowcasted incidence and isolation index during each phase of the São Paulo Plan in these cities. For all cities, we see a higher isolation in the period prior to the implementation of the plan, followed by the red (when present), orange, yellow, and green (when present) phases, respectively, showing that the phases of the plan were effective in increasing the isolation in these cities compared with less restrictive phases. On the other hand, the incidence had a specific behavior in each city. In São Paulo, it was higher prior to the implementation of the plan and during the orange phase due to the fact that the strict measures implemented prior to the plan were in response to the high incidence in this city, and the yellow and green phases were implemented only when the incidence decreased at the end of the orange phase. In Campinas, the incidence was higher in the orange and red phases, which were implemented as a response to such high incidence. In Votuporanga, the higher incidence was in the starting phases of the São Paulo Plan because of the delay of the disease in arriving there. Note that Votuporanga is an inner state city lower on the urban hierarchy compared with Campinas and São Paulo.

**Table 4:** Median of $R_{t}$, isolation index, and incidence during each phase of the São Paulo Plan in Campinas, São Paulo city, and Votuporanga

City	Phase	Median $R_{t}$	Median isolation	Median incidence
Campinas	green	0.94	24.97	1.70
	yellow	0.97	35.64	2.74
	orange	1.05	48.50	4.90
	red	0.92	50.11	4.82
	prior	1.25	69.84	1.66
São Paulo	green	1.02	30.36	1.82
	yellow	0.98	39.22	2.64
	orange	0.96	48.51	4.26
	prior	1.13	69.31	4.68
Votuporanga	yellow	1.07	18.09	4.23
	orange	1.16	24.05	4.23
	prior	0.99	34.78	2.12

Prior means the period before the São Paulo Plan was employed.

## Discussion

In this work, we explored how social isolation may have played a role in halting the spreading of the COVID-19 epidemic in the Brazilian territory. Our analysis sought to associate two temporal series, the relative social isolation index and the incidence, exploring the relationship between these two datasets in different cities in Brazil, looking for patterns and explanations in view of socioeconomical and management characteristics of each location.

The first thing that we could notice is that there is no direct and simple relationship between the social isolation index and the incidence, since the evolution of the disease is driven by multiple factors such as urban hierarchy, human development, and infrastructure development. Other studies have also encountered challenges to correlate mobility data in Brazil and the disease spread, even when other exogenous variables (temperature, humidity, and rainfall) data are included.^37^ As an example, da Silva et al.^37^ show that correlations depend strongly on the region in which the city is located, and only seldom was a significant correlation between mobility data and COVID-19 cases observed. However, even though there is no direct relationship, we identified among the metropolises some interesting relationships when we also considered the reality of each city and how it could be influencing the effectiveness of measures to stop the disease spreading. Furthermore, when we expanded the analysis to more cities and focused on the period until the peak of cases (upward phase), we were able to observe two main regimes under which isolation is effective to control the disease, which are when it is employed as a prevention or as a remedy. As discussed in Rüdiger et al.,^16^ the precise understanding of the interplay between mobility and disease spreading requires individual-level mobility data in order to take into account individual contacts and their heterogeneity. As in our work, they argue that aggregated mobility data alone are not enough to derive strong and reliable assertions to explain and predict infection behavior. However, here we were able to capture broad regimes that already allow some relevant understanding of the interplay.

We concluded that social isolation may be successfully employed to reduce the disease spread in both manners, although, when it is employed as a remedy, the isolation should be greater and the peak of the disease tends to be higher. Hence, to be really successful, isolation should be employed when the incidence is low and kept high during all the epidemic, otherwise, although delayed, an increase in the incidence eventually occurs, even though the peak will be smaller. This is in agreement with Jorge et al.,^13^ who highlighted that increasing the level of control measures only when the number of cases and hospitalizations are increasing represents a flawed strategy, unable to avert the impacts of COVID-19 on the healthcare system. Finally, we studied two special cases, when a lockdown was implemented and the São Paulo State plan, where we have some interesting findings of scenarios where isolation can be employed to stop disease spread.

There are some limitations related to the data used here. The bias of the daily isolation index is not controlled. On top of that, the daily incidence data cannot capture the asymptomatic cases. Despite these limitations, these data are known to be the best available data of the kind for epidemic management in Brazil, and it was indeed what was used by several governmental crisis committees. Finally, interesting topics for future researches revolve around trying to identify the two regimes or others where isolation may mitigate the disease spread in other datasets containing data of other locations or datasets from Brazil, but considering the period from the end of 2020 and 2021. Better understanding how social isolation may be employed to stop the spread of diseases when pharmaceuticals measures are not yet available is important not only during the COVID-19 pandemic but also in future epidemics.

## Experimental procedures

### Resource availability

#### Lead contact

Pedro S. Peixoto (ppeixoto@usp.br).

#### Materials availability

This study did not generate new unique reagents.

## Data Availability

The datasets and codes generated during this study are available at the GitHub repository Mdyn (https://github.com/pedrospeixoto/mdyn) or at Zenodo Data: https://doi.org/10.5281/zenodo.5452914)
